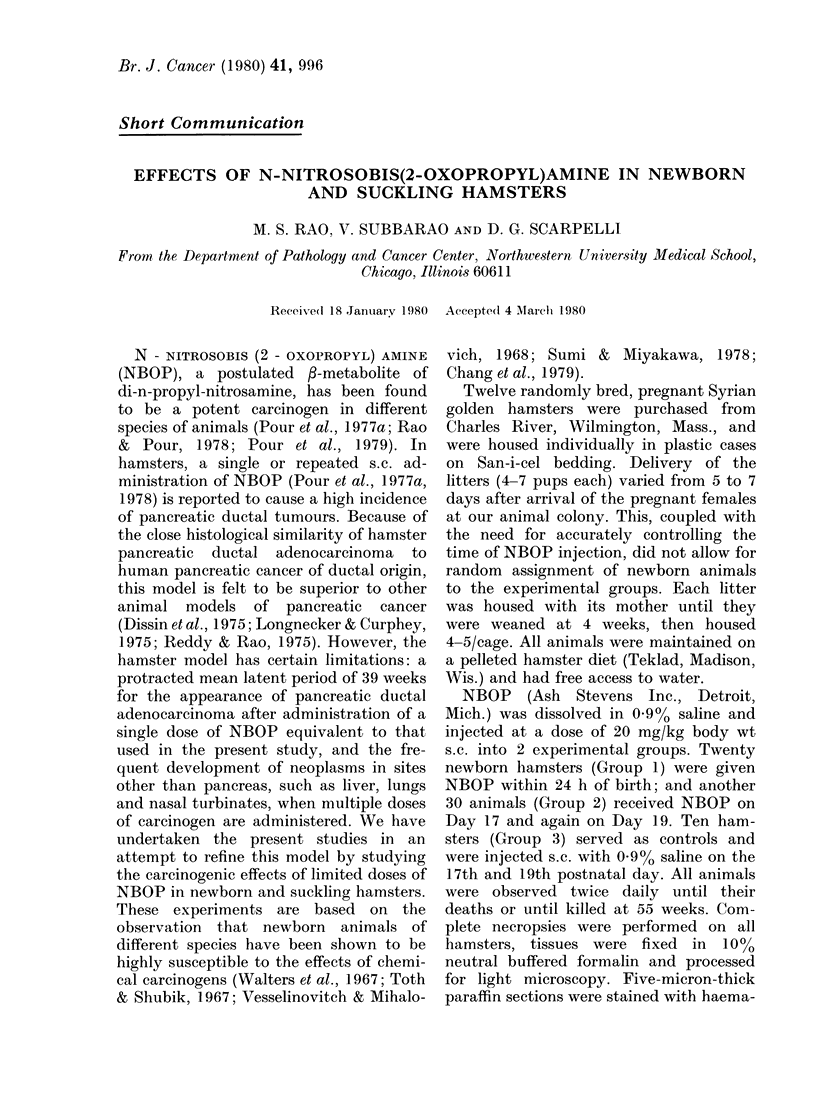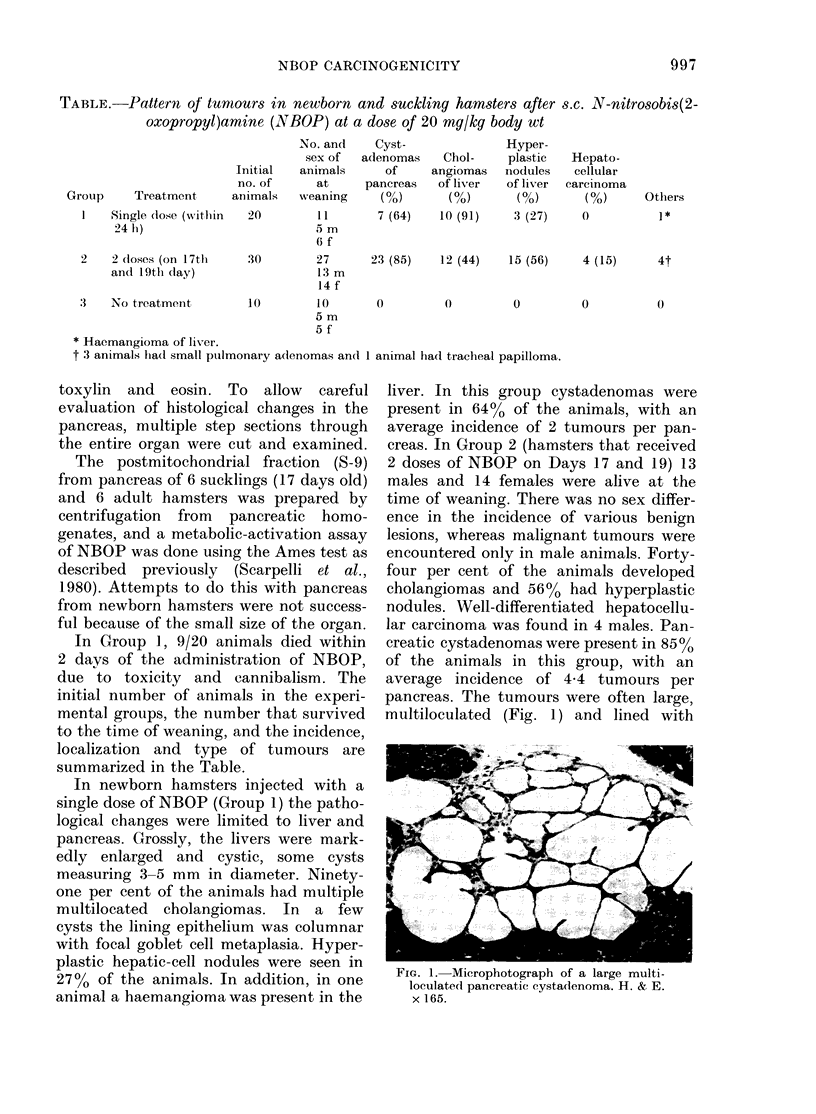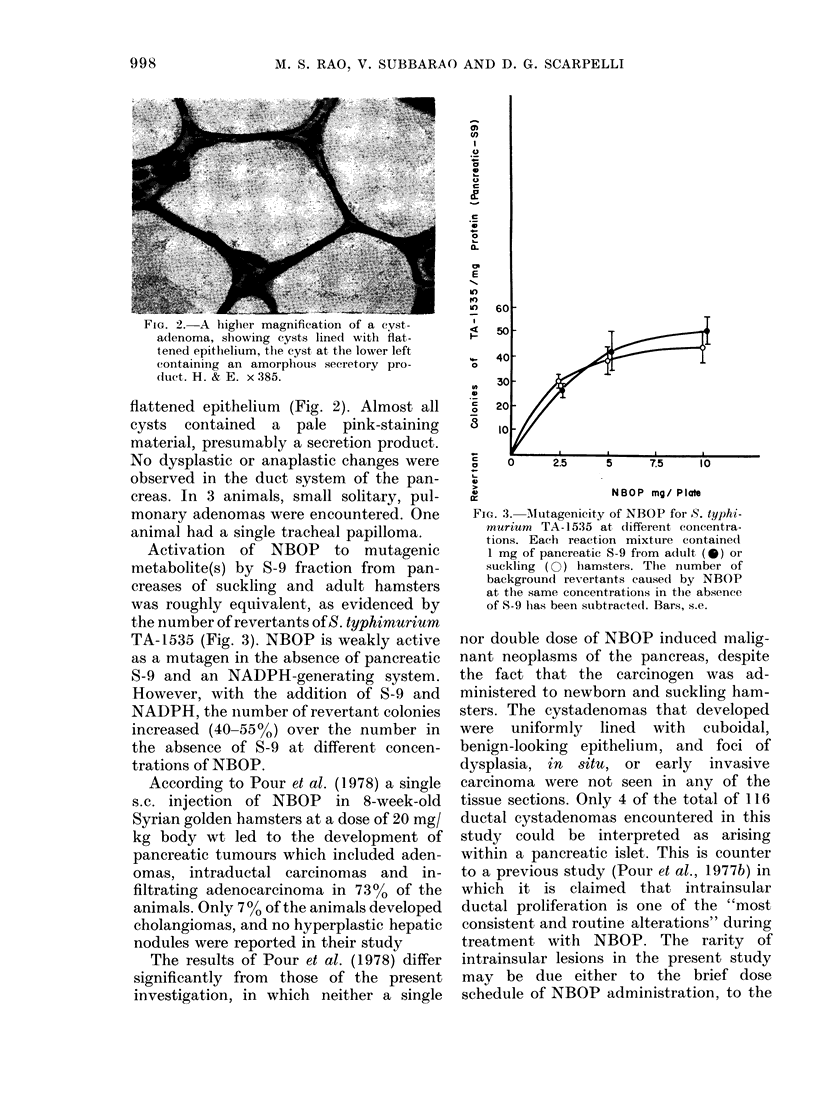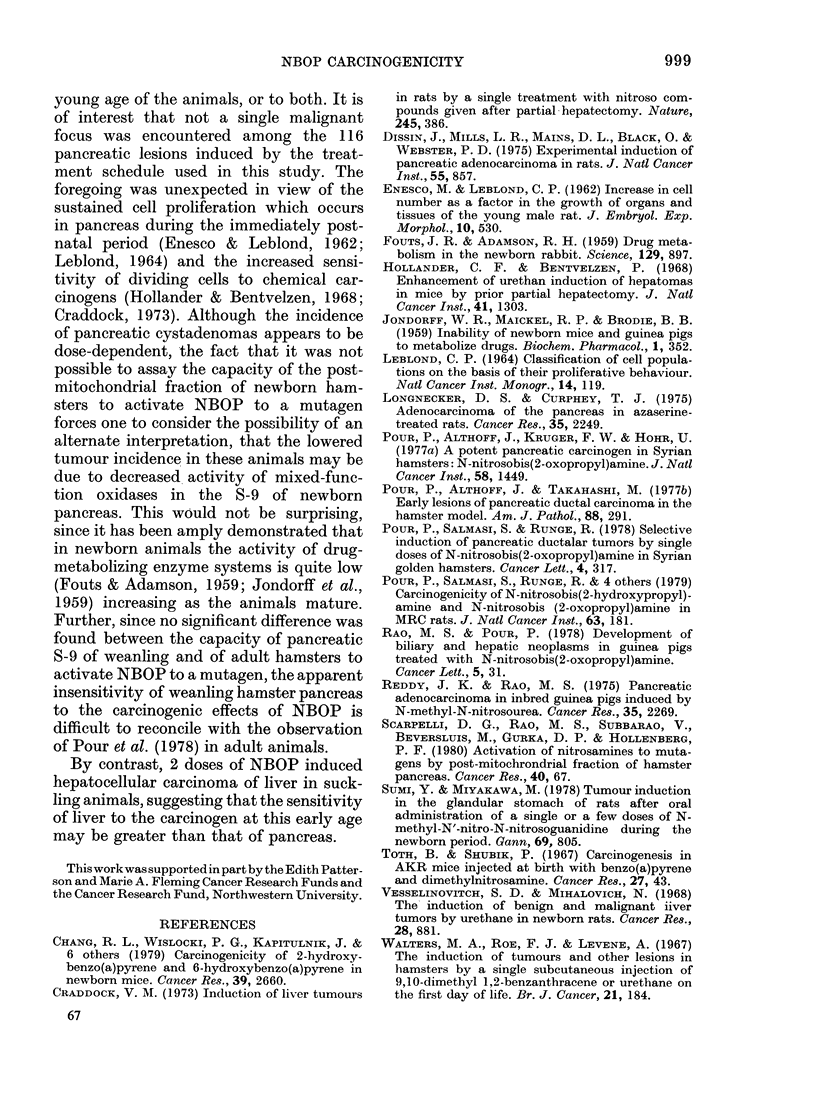# Effects of N-nitrosobis(2-oxopropyl)amine in newborn and suckling hamsters.

**DOI:** 10.1038/bjc.1980.181

**Published:** 1980-06

**Authors:** M. S. Rao, V. Subbarao, D. G. Scarpelli

## Abstract

**Images:**


*Br. J. Cancer* (1980) **41**, 996

***Short Communication***

### EFFECTS OF N-NITROSOBIS(2-OXOPROPYL)AMINE IN NEWBORN AND SUCKLING HAMSTERS

M. S. RAO, V. SUBBARAO AND D. G. SCARPELLI

*From the Department of Pathology and Cancer Center, Northwestern University Medical School, Chicago, Illinois* 60611

Received 18 January 1980 Accepted 4 March 1980

N - NITROSOBIS (2 - OXOPROPYL) AMINE (NBOP), a postulated β-metabolite of di-n-propyl-nitrosamine, has been found to be a potent carcinogen in different species of animals (Pour *et al.*, 1977*a*; Rao & Pour, 1978; Pour *et al.*, 1979). In hamsters, a single or repeated s.c. administration of NBOP (Pour *et al.*, 1977*a*, 1978) is reported to cause a high incidence of pancreatic ductal tumours. Because of the close histological similarity of hamster pancreatic ductal adenocarcinoma to human pancreatic cancer of ductal origin, this model is felt to be superior to other animal models of pancreatic cancer (Dissin *et al.*, 1975; Longnecker & Curphey, 1975; Reddy & Rao, 1975). However, the hamster model has certain limitations: a protracted mean latent period of 39 weeks for the appearance of pancreatic ductal adenocarcinoma after administration of a single dose of NBOP equivalent to that used in the present study, and the frequent development of neoplasms in sites other than pancreas, such as liver, lungs and nasal turbinates, when multiple doses of carcinogen are administered. We have undertaken the present studies in an attempt to refine this model by studying the carcinogenic effects of limited doses of NBOP in newborn and suckling hamsters. These experiments are based on the observation that newborn animals of different species have been shown to be highly susceptible to the effects of chemical carcinogens (Walters *et al.*, 1967; Toth & Shubik, 1967; Vesselinovitch & Mihalovich, 1968; Sumi & Miyakawa, 1978; Chang *et al.*, 1979).

Twelve randomly bred, pregnant Syrian golden hamsters were purchased from Charles River, Wilmington, Mass., and were housed individually in plastic cases on San-i-cel bedding. Delivery of the litters (4–7 pups each) varied from 5 to 7 days after arrival of the pregnant females at our animal colony. This, coupled with the need for accurately controlling the time of NBOP injection, did not allow for random assignment of newborn animals to the experimental groups. Each litter was housed with its mother until they were weaned at 4 weeks, then housed 4–5/cage. All animals were maintained on a pelleted hamster diet (Teklad, Madison, Wis.) and had free access to water.

NBOP (Ash Stevens Inc., Detroit, Mich.) was dissolved in 0·9% saline and injected at a dose of 20 mg/kg body wt s.c. into 2 experimental groups. Twenty newborn hamsters (Group 1) were given NBOP within 24 h of birth; and another 30 animals (Group 2) received NBOP on Day 17 and again on Day 19. Ten hamsters (Group 3) served as controls and were injected s.c. with 0·9% saline on the 17th and 19th postnatal day. All animals were observed twice daily until their deaths or until killed at 55 weeks. Complete necropsies were performed on all hamsters, tissues were fixed in 10% neutral buffered formalin and processed for light microscopy. Five-micron-thick paraffin sections were stained with haema-

TABLE.—*Pattern of tumours in newborn and suckling hamsters after s.c. N-nitrosobis(2-oxopropyl)amine (NBOP) at a dose of 20 mg/kg body wt*

| Group | Treatment | Initial no. of animals | No. and sex of animals at weaning | Cyst-adenomas of pancreas (%) | Chol-angiomas of liver (%) | Hyper-plastic nodules of liver (%) | Hepato-cellular carcinoma (%) | Others |
|---|---|---|---|---|---|---|---|---|
| 1 | Single dose (within 24 h) | 20 | 11<br>5 m<br>6 f | 7 (64) | 10 (91) | 3 (27) | 0 | 1* |
| 2 | 2 doses (on 17th and 19th day) | 30 | 27<br>13 m<br>14 f | 23 (85) | 12 (44) | 15 (56) | 4 (15) | 4† |
| 3 | No treatment | 10 | 10<br>5 m<br>5 f | 0 | 0 | 0 | 0 | 0 |

* Haemangioma of liver.

† 3 animals had small pulmonary adenomas and 1 animal had tracheal papilloma.

toxylin and eosin. To allow careful evaluation of histological changes in the pancreas, multiple step sections through the entire organ were cut and examined.

The postmitochondrial fraction (S-9) from pancreas of 6 sucklings (17 days old) and 6 adult hamsters was prepared by centrifugation from pancreatic homogenates, and a metabolic-activation assay of NBOP was done using the Ames test as described previously (Scarpelli *et al.*, 1980). Attempts to do this with pancreas from newborn hamsters were not successful because of the small size of the organ.

In Group 1, 9/20 animals died within 2 days of the administration of NBOP, due to toxicity and cannibalism. The initial number of animals in the experimental groups, the number that survived to the time of weaning, and the incidence, localization and type of tumours are summarized in the Table.

In newborn hamsters injected with a single dose of NBOP (Group 1) the pathological changes were limited to liver and pancreas. Grossly, the livers were markedly enlarged and cystic, some cysts measuring 3–5 mm in diameter. Ninety-one per cent of the animals had multiple multilocated cholangiomas. In a few cysts the lining epithelium was columnar with focal goblet cell metaplasia. Hyperplastic hepatic-cell nodules were seen in 27% of the animals. In addition, in one animal a haemangioma was present in the liver. In this group cystadenomas were present in 64% of the animals, with an average incidence of 2 tumours per pancreas. In Group 2 (hamsters that received 2 doses of NBOP on Days 17 and 19) 13 males and 14 females were alive at the time of weaning. There was no sex difference in the incidence of various benign lesions, whereas malignant tumours were encountered only in male animals. Forty-four per cent of the animals developed cholangiomas and 56% had hyperplastic nodules. Well-differentiated hepatocellular carcinoma was found in 4 males. Pancreatic cystadenomas were present in 85% of the animals in this group, with an average incidence of 4·4 tumours per pancreas. The tumours were often large, multiloculated (Fig. 1) and lined with

FIG. 1.—Microphotograph of a large multi-loculated pancreatic cystadenoma. H. & E. ×165.

FIG. 2.—A higher magnification of a cystadenoma, showing cysts lined with flattened epithelium, the cyst at the lower left containing an amorphous secretory product. H. & E. ×385.

FIG. 3.—Mutagenicity of NBOP for *S. typhimurium* TA-1535 at different concentrations. Each reaction mixture contained 1 mg of pancreatic S-9 from adult (●) or suckling (○) hamsters. The number of background revertants caused by NBOP at the same concentrations in the absence of S-9 has been subtracted. Bars, s.e.

flattened epithelium (Fig. 2). Almost all cysts contained a pale pink-staining material, presumably a secretion product. No dysplastic or anaplastic changes were observed in the duct system of the pancreas. In 3 animals, small solitary, pulmonary adenomas were encountered. One animal had a single tracheal papilloma.

Activation of NBOP to mutagenic metabolite(s) by S-9 fraction from pancreases of suckling and adult hamsters was roughly equivalent, as evidenced by the number of revertants of *S. typhimurium* TA-1535 (Fig. 3). NBOP is weakly active as a mutagen in the absence of pancreatic S-9 and an NADPH-generating system. However, with the addition of S-9 and NADPH, the number of revertant colonies increased (40–55%) over the number in the absence of S-9 at different concentrations of NBOP.

According to Pour *et al.* (1978) a single s.c. injection of NBOP in 8-week-old Syrian golden hamsters at a dose of 20 mg/kg body wt led to the development of pancreatic tumours which included adenomas, intraductal carcinomas and infiltrating adenocarcinoma in 73% of the animals. Only 7% of the animals developed cholangiomas, and no hyperplastic hepatic nodules were reported in their study

The results of Pour *et al.* (1978) differ significantly from those of the present investigation, in which neither a single nor double dose of NBOP induced malignant neoplasms of the pancreas, despite the fact that the carcinogen was administered to newborn and suckling hamsters. The cystadenomas that developed were uniformly lined with cuboidal, benign-looking epithelium, and foci of dysplasia, *in situ*, or early invasive carcinoma were not seen in any of the tissue sections. Only 4 of the total of 116 ductal cystadenomas encountered in this study could be interpreted as arising within a pancreatic islet. This is counter to a previous study (Pour *et al.*, 1977*b*) in which it is claimed that intrainsular ductal proliferation is one of the "most consistent and routine alterations" during treatment with NBOP. The rarity of intrainsular lesions in the present study may be due either to the brief dose schedule of NBOP administration, to the young age of the animals, or to both. It is of interest that not a single malignant focus was encountered among the 116 pancreatic lesions induced by the treatment schedule used in this study. The foregoing was unexpected in view of the sustained cell proliferation which occurs in pancreas during the immediately postnatal period (Enesco & Leblond, 1962; Leblond, 1964) and the increased sensitivity of dividing cells to chemical carcinogens (Hollander & Bentvelzen, 1968; Craddock, 1973). Although the incidence of pancreatic cystadenomas appears to be dose-dependent, the fact that it was not possible to assay the capacity of the post-mitochondrial fraction of newborn hamsters to activate NBOP to a mutagen forces one to consider the possibility of an alternate interpretation, that the lowered tumour incidence in these animals may be due to decreased activity of mixed-function oxidases in the S-9 of newborn pancreas. This would not be surprising, since it has been amply demonstrated that in newborn animals the activity of drug-metabolizing enzyme systems is quite low (Fouts & Adamson, 1959; Jondorff *et al.*, 1959) increasing as the animals mature. Further, since no significant difference was found between the capacity of pancreatic S-9 of weanling and of adult hamsters to activate NBOP to a mutagen, the apparent insensitivity of weanling hamster pancreas to the carcinogenic effects of NBOP is difficult to reconcile with the observation of Pour *et al.* (1978) in adult animals.

By contrast, 2 doses of NBOP induced hepatocellular carcinoma of liver in suckling animals, suggesting that the sensitivity of liver to the carcinogen at this early age may be greater than that of pancreas.

This work was supported in part by the Edith Patterson and Marie A. Fleming Cancer Research Funds and the Cancer Research Fund, Northwestern University.

### REFERENCES

Chang, R. L., Wislocki, P. G., Kapitulnik, J. & 6 others (1979) Carcinogenicity of 2-hydroxybenzo(a)pyrene and 6-hydroxybenzo(a)pyrene in newborn mice. *Cancer Res.*, **39**, 2660.

Craddock, V. M. (1973) Induction of liver tumours in rats by a single treatment with nitroso compounds given after partial hepatectomy. *Nature*, **245**, 386.

Dissin, J., Mills, L. R., Mains, D. L., Black, O. & Webster, P. D. (1975) Experimental induction of pancreatic adenocarcinoma in rats. *J. Natl Cancer Inst.*, **55**, 857.

Enesco, M. & Leblond, C. P. (1962) Increase in cell number as a factor in the growth of organs and tissues of the young male rat. *J. Embryol. Exp. Morphol.*, **10**, 530.

Fouts, J. R. & Adamson, R. H. (1959) Drug metabolism in the newborn rabbit. *Science*, **129**, 897.

Hollander, C. F. & Bentvelzen, P. (1968) Enhancement of urethan induction of hepatomas in mice by prior partial hepatectomy. *J. Natl Cancer Inst.*, **41**, 1303.

Jondorff, W. R., Maickel, R. P. & Brodie, B. B. (1959) Inability of newborn mice and guinea pigs to metabolize drugs. *Biochem. Pharmacol.*, **1**, 352.

Leblond, C. P. (1964) Classification of cell populations on the basis of their proliferative behaviour. *Natl Cancer Inst. Monogr.*, **14**, 119.

Longnecker, D. S. & Curphey, T. J. (1975) Adenocarcinoma of the pancreas in azaserine-treated rats. *Cancer Res.*, **35**, 2249.

Pour, P., Althoff, J., Kruger, F. W. & Hohr, U. (1977*a*) A potent pancreatic carcinogen in Syrian hamsters: N-nitrosobis(2-oxopropyl)amine. *J. Natl Cancer Inst.*, **58**, 1449.

Pour, P., Althoff, J. & Takahashi, M. (1977*b*) Early lesions of pancreatic ductal carcinoma in the hamster model. *Am. J. Pathol.*, **88**, 291.

Pour, P., Salmasi, S. & Runge, R. (1978) Selective induction of pancreatic ductalar tumors by single doses of N-nitrosobis(2-oxopropyl)amine in Syrian golden hamsters. *Cancer Lett.*, **4**, 317.

Pour, P., Salmasi, S., Runge, R. & 4 others (1979) Carcinogenicity of N-nitrosobis(2-hydroxypropyl)-amine and N-nitrosobis (2-oxopropyl)amine in MRC rats. *J. Natl Cancer Inst.*, **63**, 181.

Rao, M. S. & Pour, P. (1978) Development of biliary and hepatic neoplasms in guinea pigs treated with N-nitrosobis(2-oxopropyl)amine. *Cancer Lett.*, **5**, 31.

Reddy, J. K. & Rao, M. S. (1975) Pancreatic adenocarcinoma in inbred guinea pigs induced by N-methyl-N-nitrosourea. *Cancer Res.*, **35**, 2269.

Scarpelli, D. G., Rao, M. S., Subbarao, V., Beversluis, M., Gurka, D. P. & Hollenberg, P. F. (1980) Activation of nitrosamines to mutagens by post-mitochrondrial fraction of hamster pancreas. *Cancer Res.*, **40**, 67.

Sumi, Y. & Miyakawa, M. (1978) Tumour induction in the glandular stomach of rats after oral administration of a single or a few doses of N-methyl-N′-nitro-N-nitrosoguanidine during the newborn period. *Gann*, **69**, 805.

Toth, B. & Shubik, P. (1967) Carcinogenesis in AKR mice injected at birth with benzo(a)pyrene and dimethylnitrosamine. *Cancer Res.*, **27**, 43.

Vesselinovitch, S. D. & Mihalovich, N. (1968) The induction of benign and malignant iiver tumors by urethane in newborn rats. *Cancer Res.*, **28**, 881.

Walters, M. A., Roe, F. J. & Levene, A. (1967) The induction of tumours and other lesions in hamsters by a single subcutaneous injection of 9,10-dimethyl 1,2-benzanthracene or urethane on the first day of life. *Br. J. Cancer*, **21**, 184.